# Allosteric properties of mammalian ALOX15 orthologs

**DOI:** 10.1016/j.jbc.2026.111244

**Published:** 2026-02-05

**Authors:** Jiaxing Yang, Astrid Borchert, Hartmut Kuhn

**Affiliations:** Department of Biochemistry, Charité – University Medicine Berlin, Corporate Member of Freie Universität Berlin and Humboldt Universität zu Berlin, Berlin, Germany

**Keywords:** eicosanoids, lipoxygenase, lipid peroxidation, inflammation, neurodegeneration, atherosclerosis, cancer

## Abstract

Lipoxygenases (arachidonic acid lipoxygenase [ALOX]) are non-heme iron–containing dioxygenases that catalyze the oxygenation of polyenoic fatty acid–containing lipids to their corresponding hydroperoxy derivatives. These enzymes are widely distributed in highly developed plants and animals. In bacteria, they rarely occur, but they have not been detected in archaea and viruses. The human genome involves six functional *ALOX* genes (*ALOX15*, *ALOX15B*, *ALOX12*, *ALOX12B*, *ALOXE3*, and *ALOX5*) encoding for six different isoenzymes. The mouse genome carries an orthologous gene for each human *ALOX* gene, but in addition, an *Aloxe12* gene has been identified in this species. The application of isoenzyme-specific loss-of-function strategies suggested that the coding multiplicity may not be interpreted as a sign of functional redundancy. In fact, the different isoenzymes apparently fulfill different biological functions. Mammalian ALOX15 orthologs are allosteric enzymes, but the molecular basis for their allosteric properties remains controversial. In fact, two alternative hypotheses (the presence of allosteric binding sites at enzyme monomers *versus* ALOX15 dimers consist of an allosteric and a catalytic monomer) have been introduced, and this review is aimed at critically evaluating the pros and cons of these two mechanistic scenarios.

## Lipoxygenases are lipid-peroxidizing enzymes

Mammalian arachidonic acid (AA) lipoxygenases (ALOX-isoenzymes) are lipid dioxygenases (1, 2, 3), which catalyze the bimolecular reaction of polyunsaturated fatty acid (PUFA)–containing lipids with molecular dioxygen. They occur in prokaryotic and eukaryotic organisms (4, 5, 6, 7, 8) and their reaction mechanism is highly conserved. Mammalian ALOX isoenzymes carry a non-heme iron ion as a catalytically active transition metal (9) but some fungal ALOX-isoenzymes harbor a manganese ion instead (10, 11). ALOX isoenzymes are moonlighting proteins. In addition to their oxygenase activities, they also exhibit hydroperoxide isomerase and leukotriene synthase activities (12).

The human genome involves six functional *ALOX* genes, several corrupted *ALOX* pseudogenes, and an *ALOX12* antisense gene with unknown biological relevance (13). The functional *ALOX* genes encode for six different isoenzymes, which exhibit different catalytic properties. Except for the *ALOX5* gene, which is located on the long arm of chromosome 10 (Table 1), all other *ALOX* genes have been mapped to an *ALOX* gene cluster that is localized on the short arm of chromosome 17. The genes are located on both, the plus or the minus strands (Table 1). The *ALOX5* gene (71.9 kbp) is the biggest human *ALOX* gene, and the sizes of the other *ALOX* genes vary between 10 and 23 kbp. In the mouse genome, seven functional *Alox* genes have been identified. For each human *ALOX* gene, there is a single copy of orthologous gene in the mouse reference genome (*Alox15*, *Alox15B*, *Alox12*, *Alox12B*, *Alox5*, and *Aloxe3*), but in addition, a functional *Aloxe12* gene was discovered in this species. This *Aloxe12* gene, which is also a constituent of the *Alox* gene cluster on mouse chromosome 11, encodes a functional enzyme, which is highly homologous to mouse Alox15. Unfortunately, *Aloxe12* knockout mice have not been created, and thus, the biological relevance of this protein could not be explored in detail. In the human genome, the *ALOXE12* gene is a functionless pseudogene. Gene-specific knockout studies of other *Alox* genes suggested that the coding multiplicity of ALOX-isoenzymes may not be considered as a sign of functional redundancy. In fact, most human ALOX isoenzymes exhibit different catalytic properties. Moreover, *Alox15*^−/−^ mice (14) have a different phenotype than *Alox12*^−/−^ (15), *Alox5*^−/−^ (16), *Alox12b*^−/−^ (17) and *Aloxe3*^−/−^ (18) mice. Taken together, these data suggest that each ALOX-isoenzyme may fulfill isoenzyme-specific biological function. In other words, functional defects induced by inactivation of a single *Alox* gene cannot be fully compensated by upregulation of the expression of other ALOX isoenzymes.

**Table 1:** Functional human *ALOX* genes and their chromosomal localization

Name	Gene	Localization, size, range	Former names	Major expression sites in	Coding strand	Discovered	Reference
ALOX12	*ALOX12*	17p13.1, 14.7 kbp, 6,996,049–7,010,754	pl12-LOX^a^	Blood platelets, megakaryocytes, skin	Plus	1974–75	(27, 28)
ALOX15	*ALOX15*	17p13.2, 11.4 kbp, 4,630,919–4,642,294	15-LOX, 12/15-LOX, 15-LO, lc12-LOX^b^	Eosinophils, airway epithelium, alternatively activated monocytes	Minus	1975	(32)
ALOX5	*ALOX5*	10q11.21, 71.9 kbp, 45,374,216–45,446,117	5-LOX, 5-LO	Leukocytes, dendritic cells	Plus	1976	(43)
ALOXE3	*ALOXE3*	17p13.1, 23.0 kbp, 8,095,900–8,118,916	eLOX3	Keratinocytes, corneocytes, epidermis	Minus	1995–97	(47, 48, 49)
ALOX15B	*ALOX15B*	17p13.1, 10.1 kbp, 8,039,034–8,049,134	15-LOX2, 15-LO2	Hair roots, skin, prostate epithelium, ovary	Plus	1997–98	(50, 56)
ALOX12B	*ALOX12B*	17p13.1, 15.1 kbp, 8,072,636–8,087,716	12R-LOX	Keratinocytes, skin, tonsils, respiratory epithelium	Minus	1998	(56, 57, 59)

The human genome involves six functional *ALOX* genes, and except for the *ALOX5* gene (localized on chromosome 10), all other human ALOX genes have been mapped to the *ALOX* gene cluster localized on the short arm of chromosome 17. In the table, the enzymes are ordered according to the date of first description, and corresponding references are given. Protein coding strands are indicated. Several ALOX isoenzymes were first described in species other than humans.

a Platelet-type 12-LOX.

b Leucocyte-type 12-LOX.

## Historical breakdown of ALOX research

Lipoxygenases were first described in the mid-1930s in soybean seeds, and initially, the protein(s) were called lipoxidase(s) (19). In 1947, the dominant enzyme (soybean LOX1) was purified to homogeneity and was crystallized (20, 21), but it took more than 40 years to solve its crystal structure (22, 23). The initial X-ray coordinates were later on refined to a molecular resolution of 1.4 Å (24) and today, soybean LOX1 is one of the best characterized lipoxygenase isoenzyme. Unfortunately, its biological function is still a matter of discussion, and it remains unclear why this enzyme occurs in such large quantities in soybean seeds.

In animal tissues, the presence of true lipoxygenases has long been challenged. In fact, lipid peroxidation in animal tissues has frequently been related to the catalytic activity of hemoproteins (25, 26). In 1974 (27) the first animal lipoxygenase was discovered in human thrombocytes (Table 1), and 1 year later, these findings were confirmed for blood platelets of other species (28). Today, this enzyme is known as ALOX12, and it has been implicated in the regulation of hemostasis (15, 29) and in skin development (30). Recently, the 3D structure of recombinant human ALOX12 has been solved by cryo-electron microscopy (31). According to these data, the single polypeptide chain of human ALOX12 folds into the typical two-domain ALOX structure (31) but in aqueous solutions, the enzyme tends to oligomerize (31).

In 1975 a different animal lipoxygenase was discovered in rabbit reticulocytes (Table 1). This enzyme was capable of oxygenating not only free polyenoic fatty acids (PUFAs) but also phospholipids carrying such structural components (32). This enzyme, which is today known as rabbit ALOX15, also occurs in reticulocytes of other mammals (33) including humans (34, 35). It is constitutively expressed at high levels not only in immature red blood cells, eosinophils, and airway epithelial cells (35) but also in alternatively differentiated monocytes and macrophages (36, 37, 38). Rabbit ALOX15 was purified to homogeneity in 1979 (39) and at that time, it was the only animal ALOX isoenzyme that was available as pure protein. In 1990, the enzyme was first crystallized (40) but its 3D structure (2.4 Å) was only solved 7 years later (41). Unfortunately, the original X-ray dataset was incomplete (41), but reinterpretation of the original atomic coordinates defined two different protein conformations (42). One structural conformer carried an inhibitor and adopted a closed conformation, in which the entrance into the putative substrate-binding pocket was blocked. In contrast, the inhibitor-free conformer involved an open substrate-binding pocket, which allowed substrate binding. This 3D model suggested a high degree of structural flexibility of the enzyme molecule and provided evidence for substantial structural rearrangement of the enzyme protein upon ligand binding (42).

In 1976, enzyme(s) were detected in rabbit polymorphonuclear leukocytes (Table 1), which converted exogenously added AA to 5*S*-hydroxy-6,8,11,14-eicosatetraenoic acid. The formation of this metabolite was not inhibited by the prostaglandin synthase (PTGS) inhibitor indomethacin, and thus, the presence of a lipoxygenase was concluded (43). Today, this enzyme is known as rabbit ALOX5. The enzyme was later on purified from native and recombinant sources to electrophoretic homogeneity (44, 45) and has been well characterized with respect to its protein-chemical, molecular biological, and functional properties (1, 3). Studying the functional stability of the human enzyme ortholog, an internal “destabilizing sequence” was detected. After mutation of this sequence, the protein was crystallized, and its crystal structure was solved at a molecular resolution of 2.4 Å (46). In this structure, the putative substrate-binding pocket was identified as an elongated internal cavity with no clear access to solvent. The bulky side chains of Phe177 and Tyr181 (FY-cork) seal this cavity at one end, preventing inward diffusion of solvent and ligands. The opposite end of this pocket was blocked by the side chain of Trp147. This structural scenario did of course raise the question of how substrate fatty acids may gain access to the catalytic iron, and two possible scenarios have been suggested: (i) movement of the FY-cork together with the side chain of the securing Trp599 (46). However, such opening would require simultaneous movement of three bulky side chains (Phe177, Tyr181, and Trp599). (ii) Rotamer shift of Trp147 at the opposite end of the substrate-binding pocket. Such a rotamer shift would only require the movement of a single amino acid side chain. These considerations suggest that fatty acid substrates might enter the active site of human ALOX5 from the opposite direction, as it is the case in human ALOX15B, which lacks the FY-cork (46).

In 1995 (47) a mouse genomic phage library (mouse strain 129) was screened using a human ALOX15 complementary DNA (cDNA) probe, and a novel mouse ALOX gene was identified. The corresponding cDNA (nucleotide sequence) was 68% identical with that of mouse Alox15 and 65% identical with that of mouse Alox12. Transcripts of this novel mouse Alox isoenzyme were detected in large quantities in the epidermis, and thus, the enzyme was initially called Aloxe. Today, it is known as Aloxe3 (Table 1). In parallel (48) other authors isolated a novel Alox cDNA from an RNA extract prepared from the epidermis of newborn mice. The amino acid sequence was identical to that reported (47) and the enzyme was characterized as an AA 12*S*-lipoxygenating enzyme. In a third study (49) another group screened a mouse skin papilloma cDNA library with a human ALOX12 probe and isolated a cDNA, the sequence of which was identical to that published in (47). When expressed in human embryonic kidney cells, the recombinant protein oxygenated AA to 12(S)-hydroxyeicosatetraenoic acid, and the corresponding gene was mapped to mouse chromosome 11. Taken together, these data indicate that three different groups contributed to the discovery of ALOXE3.

Human ALOX15B (Table 1), which was formerly named 15-LOX2, was first discovered in 1997 in human skin (50). The biological functions of ALOX15B are quite diverse and have recently been reviewed (51). Human ALOX15B is an AA 15-lipoxygenting enzyme, but its mouse ortholog, which was originally named Alox8, converts free AA mainly to 8*S*-hydroperoxyeicosatetraenoic acid (HpETE) (52, 53). This nomenclature is somewhat confusing since it gives the impression that mouse Alox8 belongs to another ALOX subfamily than human ALOX15B. This is, however, not the case. In fact, despite their functional differences, mouse 8-LOX and human 15-LOX2 are orthologous enzymes (they originate from a common precursor) in the two mammalian species, and thus, they should be named ALOX15B (human enzyme) and Alox15b (mouse enzyme). The AA 8*S*-lipoxygenating mouse Alox15b can easily be converted to an AA 15S-lipoxygenating enzyme by site-directed mutagenesis of two consecutive amino acids (Tyr603Asp + Hist604Val), and an inverse mutagenesis strategy on recombinant human ALOX15B partly murinized the reaction specificity of this enzyme (53). This mutagenesis worked not only for the recombinant proteins but also under *in vivo* conditions (54). Although the catalytic activity of the recombinant Tyr603Asp + Hist604Val double mutant of mouse Alox15b was somewhat lower than that of the wildtype enzyme, the formation of similar amounts of Alox15b products was observed in *ex vivo* activity assays using phorbol ester–treated tail skin of *Alox15b*-knock-in mice and wildtype control animals as enzyme source (54). In the Freunds’ adjuvant–induced skin inflammation model, *Alox15b*-knock-in mice were protected from the development of inflammatory symptoms, but no effects of this genetic manipulation of the *Alox15b* gene were observed in the dextran sodium sulfate–induced colitis model (55).

Mouse Alox12b, which was formerly named 12R-LOX, was first cloned in 1998 from a cDNA library prepared from phorbol ester–treated mouse epidermis (56). When expressed in human embryonic kidney cells, the recombinant protein converted methyl arachidonate to the corresponding 12R-HETE ester. Interestingly, neither free AA nor free linoleic acid (LA) were suitable substrates for the recombinant protein (57). The structure–function relation of this enzyme has later been explored in more detail, and Val631 was identified as a major sequence determinant for its reaction specificity (58). The human ALOX12B ortholog was also first described in 1998, and the enzyme was cloned from an RNA extract of human keratinocytes (59). The deduced amino acid sequence shared 50% identity with other human ALOX-isoenzymes, and when expressed in HELA cells, the enzyme oxygenated exogenous AA predominantly to 12*R*-HETE. These data were somewhat surprising since mouse Alox12b did not accept free AA as a substrate (57, 58). Analysis of the oxygenation product formed from [10*R*-^3^H] and [10*S*-^3^H]-labeled AAs revealed that 12*R*-HETE synthesis was associated with stereospecific removal of the pro-R hydrogen from C10 of the substrate, and this result suggests the ALOX origin of the oxygenation product (59).

## Biological functions of mammalian ALOX15 orthologs

**Figure 1:**
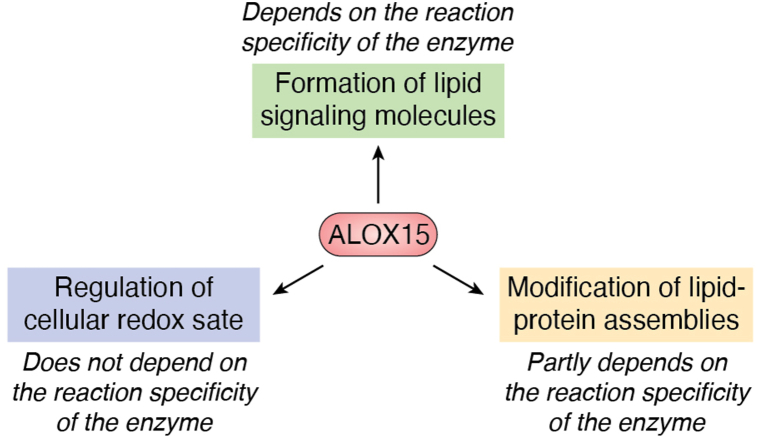
**Mechanistic principles for the biological functions of mammalian ALOX isoenzymes**. The formation of lipid signaling molecules (*green*) strongly depends on the reaction specificity of the ALOX isoenzymes. In fact, AA 12-lipoxygenating enzymes produce different patterns of oxygenation products than AA 15-lipoxygenating proteins. The regulation of the cellular redox state (*blue*) does not depend on the reaction specificity of the ALOX isoenzymes since 5-HpETE, 8-HpETE, 12-HpETE, and 15-HpETE have similar redox properties. The extent of oxidative modification of complex lipid–protein assemblies, such as biomembranes and lipoproteins (*brown*), partly depends on the reaction specificity of the ALOX isoenzymes since the membrane oxygenase activity of AA 15-lipoxygenating ALOX isoenzymes is higher than that of AA 12-lipoxygenating and AA 8-lipoxygenating enzymes. The different catalytic activities are mainly related to the high LA content of mammalian biomembranes and lipoproteins. In fact, LA is only a good substrate for AA 15-lipoxygenating ALOX isoenzymes. AA, arachidonic acid; ALOX, arachidonic acid lipoxygenase; HpETE, hydroperoxyeicosatetraenoic acid; LA, linoleic acid.

The physiological and pathophysiological functions of mammalian ALOX15 orthologs are rather complex and have been discussed in a number of previous review articles (2, 60, 61, 62). Although there are currently interesting new developments in the field (63, 64, 65, 66, 67) it would exceed the frame of this article to discuss these aspects in detail. However, we would like to briefly review the three different mechanisms by which ALOX isoenzymes fulfill their biological functions (Fig. 1).

i. Biosynthesis of signaling molecules, such as eicosanoids and related lipid mediators: This function also includes functional modification of other lipid signaling systems, such as endocannabinoids (68), diacyl glycerides (69) and sphingolipids (70). Since most human ALOX isoenzymes biosynthesize different oxygenation products, functional inactivation of the ALOX15 gene cannot be compensated by activation of the ALOX5, ALOX12, or ALOX12B pathways. If an ALOX isoenzyme exhibits its biological functions *via* this mechanism, the reaction specificity of the enzyme is of major relevance.
ii. Formation of redox-active metabolites: Since ALOX isoenzymes oxygenate PUFAs to their corresponding hydroperoxy derivatives, their catalytic activity increases the cellular oxidation potential. Thus, the catalytic activities of ALOX isoenzymes modify the activity of redox-regulated transcription factors, which alters the expression levels of redox-sensitive genes. Thus, the intracellular activity of ALOX isoenzymes changes the cellular gene expression pattern. Except for ALOXE3, which functions under physiological conditions as hydroperoxide isomerase, all other ALOX isoenzymes catalyze the formation of hydroperoxy lipids. If an ALOX isoenzyme mediates its biological functions *via* this mechanism, the reaction specificity of the enzyme may not be of functional relevance. In fact, all hydroperoxy fatty acids, such as 5-HpETE, 8-HpETE, 12-HpETE, and 15-HpETE, share similar redox properties, and thus, they modify the cellular redox state in similar ways.
iii. Structural and functional modification of complex lipid–protein assemblies: Among the human ALOX isoenzymes, ALOX15 and ALOX15B are capable of oxygenating biomembranes and lipoproteins, and these catalytic activities have been implicated in targeted degradation of intracellular organelles during cell differentiation and maturation (71, 72) as well as in the pathogenesis of atherosclerosis (73, 74). In other words, functional impairment of human ALOX15 can be compensated by upregulation of the ALOX15B pathway but not by a similar upregulation of the ALOX12, ALOX12B, or ALOXE3 pathways. In mice, upregulation of the Alox15b pathway cannot compensate for Alox15 deficiency since recombinant mouse Alox15b is not capable of oxygenating biomembranes and lipoproteins. The extent of oxidative modification of complex lipid–protein assemblies partly depends on the reaction specificity of ALOX15 orthologs since the membrane oxygenase activity of AA 15-lipoxygenating ALOX15 orthologs is higher than that of AA 12-lipoxygenating and AA 5-lipoxygenating enzymes.

## 3D structure of mammalian ALOX15 orthologs and noncovalent enzyme dimerization

**Figure 2:**
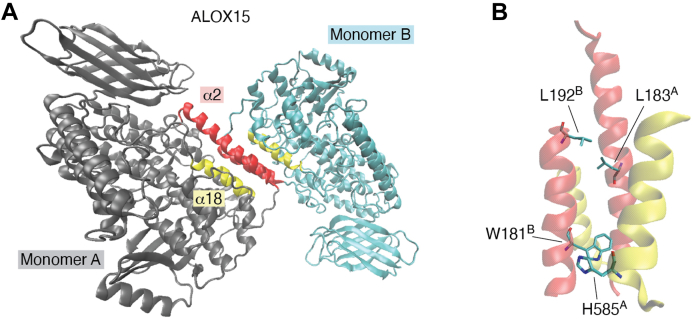
**The intermonomer interface in the crystal structure of rabbit ALOX15**. *A*, dimeric crystal structure of rabbit ALOX15 (Protein Data Bank code: 2P0M). Monomer A is shown in *gray*, and monomer B is shown in *cyan*. The α2 helices of the two monomers are labeled in *red* and α18 helices in *yellow*. Since binding of the inhibitor induced structural changes, the ALOX15 dimer represents a heterodimer. *B*, spatial arrangement of the major structural motifs that are important for intermonomer interactions. ALOX15, arachidonic acid 15-lipoxygenase.

The refined crystal structure of a rabbit ALOX15–inhibitor complex indicates the presence of two structurally distinct protein molecules in the asymmetric unit (42). In this heterodimeric complex (Protein Data Bank code: 2P0M), one monomer (monomer B) carries the inhibitor within the substrate-binding pocket, whereas the other monomer (monomer A) remains unliganded. The intermonomer interface (Fig. 2*A*), which is mainly formed by the α2 and the α18 helices of the two monomers (Fig. 2*B*), is stabilized by hydrophobic interactions. However, for a long time, it remained unclear whether enzyme dimerization was simply a consequence of enzyme crystallization or whether ALOX15 dimers may also occur in aqueous solutions. To shed light on this question, small-angle X-ray scattering studies were carried out, and the data suggested that rabbit ALOX15 in aqueous solution may undergo reversible dimerization. The relative shares of monomeric and dimeric enzymes depend on the experimental conditions (75). In fact, the degree of protein dimerization depends on enzyme concentration, temperature, pH, ionic strength, and the presence or absence of active site ligands (75, 76). Free-energy calculations on the solution structure of the rabbit ALOX15 dimer (75) suggested that a top-to-top monomer arrangement is the thermodynamically most stable dimer configuration. In this structure, monomers A and B of rabbit ALOX15 are linked together predominantly *via* the interactions of their α2 and α18 helices (Fig. 2*A*), and the following hydrophobic amino acids have been implicated in intermonomer interaction: (i) Leu183, Leu188, Leu192, and Trp181 as constituents of the α2 helix (Fig. 2*B*) and (ii) His585 as constituents of the α18 helix (Fig. 2*B*). To explore the relative contributions of some of these key amino acids, mutagenesis studies were carried out, in which the hydrophobic side chains of Trp181 and His585 (Trp181Glu + His585Glu double mutant) were replaced by charged Glu residues. Similarly, the Leu183Glu + Leu192Glu double mutant was created, and comparison of the small-angle X-ray scattering patterns of the wildtype enzyme with those of the two double mutants suggested disturbed intermonomer interactions for the mutant enzymes (76).

Other mammalian ALOX isoenzymes also exhibit a tendency for protein dimerization. Human ALOX12 is present in aqueous solutions in different oligomeric states ranging from enzyme monomers to hexamers (31). In the crystal structure (Protein Data Bank code: 7LAF) of the ALOX15B loop mutant (deletion of residues 73–79), the enzyme was also identified as a protein dimer (77). However, more recent hydrogen–deuterium exchange mass spectrometric data on recombinant human ALOX15B in aqueous solutions suggested a high degree of deuterium incorporation into the peptide 185 to 192 (77, 78). This finding suggests that the α2 helix of this enzyme may not be shielded from water by protein dimerization, and thus, ALOX15B dimerization in aqueous solutions is apparently limited. Human ALOX5 does also dimerize, but for this ALOX-isoenzyme, covalently linked enzyme dimers have been identified (79). When Cys159, Cys300, Cys416, and Cys418 were mutated to Ser, ALOX5 dimerization was largely prevented. ALOX5 dimerization was later on confirmed in another study (80). Under these conditions, ALOX5 dimerization prevented binding of the enzyme to artificial biomembrane models (nanodiscs) and inactivated the enzyme (80).

## Reaction kinetics of mammalian ALOX15 orthologs and allosteric properties

The reaction kinetics of mammalian ALOX15 orthologs are rather complex because of several reasons: (i) ALOX-isoenzymes require small amounts of hydroperoxides for enzyme activation, which is the reason for the kinetic lag phase. (ii) During lipid peroxidation, ALOX-isoenzymes undergo suicidal inactivation. (iii) The enzymes catalyze simultaneously lipoxygenase, hydroperoxide isomerase (hydroperoxidase), and leukotriene-synthase reactions, and the extent of these three reactions depends on the reaction conditions. (iv) Excess of fatty acid substrates inhibits the oxygenase reaction (substrate inhibition). Comprehensive kinetic schemes involving all these aspects are currently not available for any ALOX isoenzyme, but for rabbit ALOX15, a kinetic model has previously been worked out, which included simultaneous catalysis of LA dioxygenation and product (13S-hydroperoxyoctadecadienoic acid [HpODE]) isomerization (81). This model was based on the assumption that the substrates for the oxygenase reaction (polyenoic fatty acids) and for hydroperoxide isomerization (13S-HpODE) compete for the same binding site of the monomeric enzyme. The rate constants for the different elementary reactions of LA oxygenation were calculated, and these data confirmed that hydrogen abstraction from the bisallylic methylene of the substrate fatty acid is the rate-limiting step of the oxygenase reaction (81). Furthermore, the dissociation constants for the ALOX15–LA complex (*K*_*m*S_, 8.8 μM), the ALOX15–13S-HpODE complex (*K*_*m*P_, 2.0 μM), and the ALOX15–LA-O_2_ complex (*K*_*m*O_, 3.7 μM) were calculated (81). Taken together, these data prompted the following conclusions (81): (i) LA and 13S-HpODE compete with each other for binding at the active site of the enzyme. (ii) When LA is present in large amounts, the oxygen concentration determines the relative shares of dioxygenase and hydroperoxidase activities of the enzyme (81). (iii) The affinity of the ALOX15–LA complex for oxygen is rather high, and this finding was later on confirmed by detailed kinetic measurements using other ALOX-isoenzymes (82).

**Figure 3:**
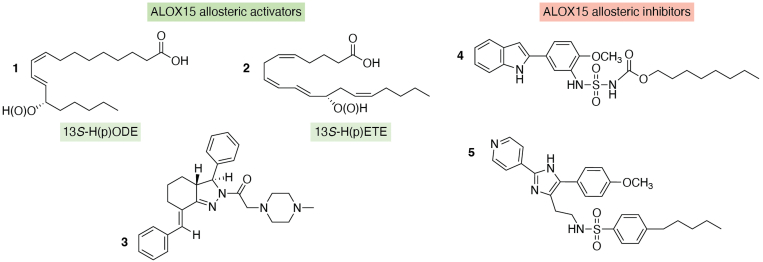
**Selected allosteric effectors of mammalian ALOX15 orthologs. 1** to **3**, allosteric ALOX15 activators. **4**, **5**, allosteric ALOX15 inhibitors. **1**, 13S-H(p)ODE; **2**, 12S-H(p)ETE. ALOX, arachidonic acid lipoxygenase; (p)ETE, hydro(peroxy)eicosatetraenoic acid; H(p)ODE, hydro(peroxy)octadecadienoic acid.

Unfortunately, when this kinetic model was worked out, the allosteric properties of ALOX15 orthologs had not been described, and thus, the potential role of allosteric effectors was not included in this model. However, today we know that mammalian ALOX-isoenzymes are allosteric enzymes, and the chemical structures of selected allosteric regulators are shown in Figure 3. 13*S*-hydro(peroxy)octadecadienoic acid and 12*S*-hydro(peroxy)eicosatetraenoic acid, the major products of LA and AA oxygenation catalyzed by mammalian ALOX15 orthologs, modified the kinetic properties of human ALOX15 (83, 84). In fact, the addition of the two compounds modified the [*k*_cat_/*K*_*M*_]^AA^/[*k*_cat_/*K*_*M*_]^LA^ ratio in similar ways, and these data suggest that these two compounds may function as allosteric activators. Similar results were obtained for human ALOX15B (83) and human ALOX12 (85). The bicyclic pyrazoline 3 (86, 87) has also been identified as activator of human ALOX15 (88). This compound did not affect *K*_*M*_ but strongly increased *k*_cat_ for both AA and LA oxygenation. Since this compound did neither compete with other active site ligands nor with substrate-derived effectors (88), a separate activator binding site (allosteric binding site) at the ALOX15 protein was suggested, which is different from the catalytic center of the enzyme.

For several ALOX15 inhibitors (compounds **4** and **5** in Fig. 3), substrate-specific inhibition was reported (89, 90). These compounds effectively inhibited ALOX15-catalyzed LA oxygenation in the nanomolar range, but AA oxygenation was not affected at these concentrations. In fact, more detailed investigations indicated that the IC_50_ values of these compounds for AA and LA oxygenation differed by several orders of magnitude. These findings were unexpected, and considering the previous observation that both LA and AA bind at the active site of the mammalian ALOX15 orthologs with comparable affinities, the different IC_50_ values suggested that the mechanism of lipoxygenase catalysis is more complex than previously anticipated. Specifically, when LA was used as the substrate, compound **4** induced a marked decrease in k_*cat*_, whereas the *K*_*M*_ values remained largely unaffected (89). In contrast, for AA oxygenation, a decrease in both *k*_cat_ and *K*_*M*_ values was observed. More detailed evaluation of the kinetic data indicated a noncompetitive mode of inhibition for LA oxygenation with a *K*_*i*_ of 21.9 ± 0.5 nM and a noncompetitive mode of inhibition with a *K*_*i*_ of 0.95 ± 0.06 μM for AA oxygenation. Taken together, these results suggested that the binding of LA as an oxygenation substrate and of the inhibitor occurs independently at different sites. In contrast, when AA is used as an oxygenation substrate, the inhibitor predominantly binds to the enzyme–substrate complex (89). Taken together, these kinetic data suggest that mammalian ALOX15 orthologs are allosteric enzymes, but the molecular basis of the allosteric properties remains a matter of discussion.

## Structural basis of the allosteric properties of mammalian ALOX15 orthologs

**Figure 4:**
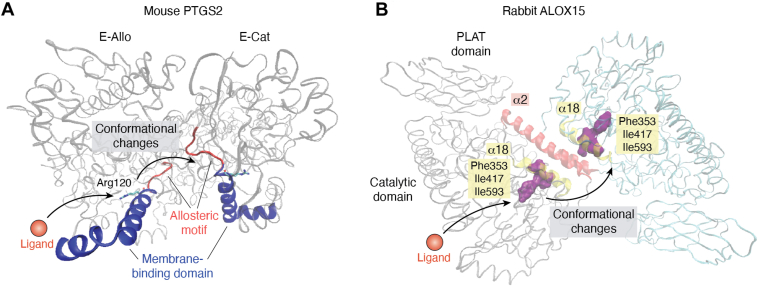
**Intermonomer crosstalk in the dimers of mouse PTGS2 and rabbit ALOX15**. *A*, the membrane-binding domain of each PTGS2 monomer within the enzyme dimer is shown in *blue*, and the allosteric motif is shown in *orange*. Ligand binding at the allosteric monomer (E-Allo), which involves contact of the ligand with Arg120, induces movement of this structural motif (residues 123–129), and these conformational alterations are subsequently translated to the binding cavity of the catalytic monomer (E-Cat). *B*, in the ground-state ALOX15 dimer, the two monomers are associated *via* α2 (*orange*) and α18 (*yellow*) helices. Phe353, I418, and Ile593 (Ile593 is part of the α18 helix) form the bottom of the substrate-binding cavity. Ligand binding in the substrate-binding pocket of one monomer induces conformational changes in the intermonomer interface of the ALOX15 dimer, which may further be translated to the substrate-binding pocket of the other monomer. ALOX, arachidonic acid lipoxygenase; PTGS, prostaglandin synthase.

Allostery can best be described as a phenomenon in which ligand binding at one site of a catalytically active protein structurally modifies other regions of the same or of an attached macromolecule (91). In principle, all nonfibrous proteins exhibit allosteric properties, but structural identification of functional allosteric-binding sites is a challenging task. There are computer-assisted prediction tools for the identification of potential allosteric-binding sites (92, 93) but experimental confirmation of their functionality is not always straightforward. Mammalian ALOX15 orthologs are allosteric enzymes, and there are currently two mechanistic scenarios to explain the allosteric properties of these enzymes.

i. The presence of binding sites for allosteric ligands outside the substrate-binding pocket of ALOX15 monomers. By employing *in silico* prediction tools, three different allosteric-binding sites were identified in human ALOX15, and one of them was interconnected with the substrate-binding pocket (86). For this allosteric-binding site, low–molecular weight ligands were developed, and some of them activated but others inactivated the catalytic activity of the enzyme (86, 87). To answer the question of why ligands binding at the same allosteric center induce either activation or inhibition of the enzyme, the authors performed a number of kinetic studies, and the results suggested that the allosteric activators elevated the catalytic activity of the enzymes by reducing the degree of substrate inhibition. The inhibitory ligands may also prevent substrate inhibition, but at the same time, they attenuate turnover numbers (87). Subsequent molecular dynamics (MD) simulations suggested that activating allosteric ligands prevents ALOX15 from being shifted into a “catalytically inactive conformation.” In contrast, inhibitory ligands restrain the mobility of active site amino acids and reduce the structural flexibility of the enzyme that is needed for effective substrate binding. Unfortunately, it has not been described in detail how the “catalytically inactive conformation” of the enzyme looks like and which amino acid side chains may play a critical role for structural flexibility. Another example (94) of an allosteric ligand that binds outside the substrate-binding pocket of human ALOX15 is 3-O-acetyl-11-keto-beta-boswellic acid (AKBA). This naturally occurring anti-inflammatory compound (95) was hypothesized to allosterically activate human ALOX15 by docking into a crevice localized between the N-terminal beta-barrel domain and the C-terminal catalytic subunit of the enzyme. AKBA was suggested to ionically interact with Arg98 that is localized in the N-terminal beta-barrel domain. The Arg98Ala exchange prevented activation of the enzyme in a cellular assay system. Prevention of allosteric activation of the enzyme is certainly one way to explain the experimental data (94) but in cellular systems, there are alternative explanations. In fact, the molecular basis for AKBA-induced activation of ALOX15 has not been explored in detail, and it remains unclear how exactly AKBA binding improves the catalytic activity of the enzyme. Moreover, *in vitro* activity assays with recombinant human ALOX15 have not been carried out, and thus, the allosteric character of enzyme activation has not been proven. Finally, the stability of the ALOX15–AKBA complex has not been tested, and it remains unclear whether this complex is catalytically productive.
ii. After protein dimerization, the substrate-binding pocket of one monomer may serve as binding site for allosteric ligands. This concept was introduced by Bill Smith for PTGSs (96, 97) and it is nicely reviewed by Dong and Malkowski in the present issue. In the absence of substrate, PTGS-isoenzymes (PTGS1, PTGS2) are present in aqueous solutions as structural homodimers, but they may function as heterodimers (98). To initiate catalysis, an allosteric ligand may bind in the substrate-binding pocket of one monomer of the PTGS dimer. This monomer then functions as allosteric subunit, and ligand binding may induce a conformational change in the partner monomer (catalytic monomer) of the enzyme dimer (Fig. 4*A*). These ligand-induced conformational changes improve the affinity of the catalytic monomer for substrate fatty acids. The efficiency of AA oxygenation at the catalytic monomer depends on the chemical structure of the ligand bound in the substrate-binding pocket of the allosteric monomer, and both activating and inhibiting effects are possible (98). The functionality of the two monomers within a PTGS dimer is interchangeable (99). In fact, double-quantum coherence spectroscopy data indicated that tyrosyl radicals, which are formed during the oxygenation of AA to PGG_2_, were generated in both monomers of the PTGS2 dimer (99) and fluorine magnetic resonance spectroscopy provided a deeper insight into the conformational changes associated with allosteric regulation of PTGS isoenzymes (100, 101).

**Figure 5:**
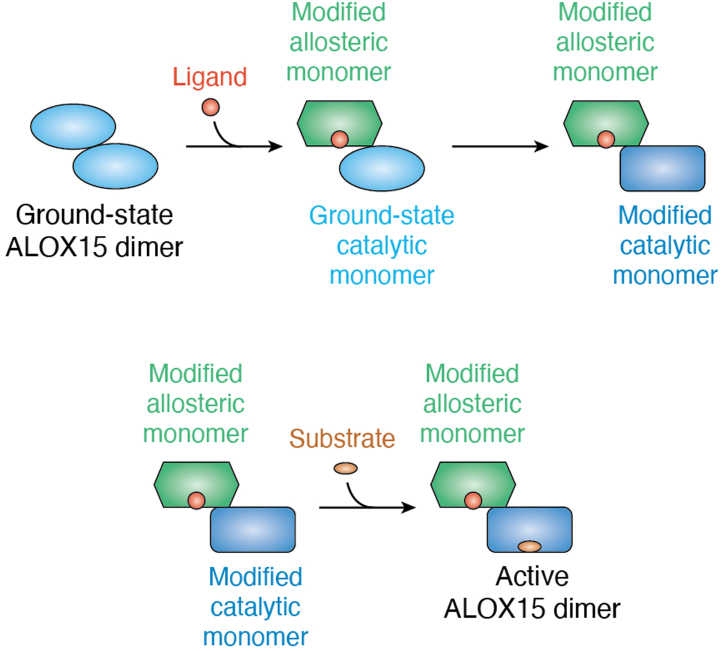
**Schematic representation of the dimeric mechanism of allosteric regulation of mammalian ALOX15 orthologs**. In aqueous solutions, the ground-state ALOX15 dimer consists of two structurally identical monomers. Binding of an allosteric ligand in the substrate-binding pocket of one monomer induces conformational changes in this monomer (modified allosteric monomer) and defines it as an allosteric monomer. It does not play any role which of the two monomers accepts the ligand. The ligand-induced conformational changes are then translated *via* the intermonomer interface to the catalytic monomer and induce structural alterations in this monomer (modified catalytic monomer). These structural changes improve or inhibit substrate binding and thus increase or reduce the rate of substrate oxygenation (active ALOX15 dimer). Thus, this allosteric mechanism involves conformational alterations in both the allosteric and catalytic monomers. ALOX15, arachidonic acid 15-lipoxygenase.

Since mammalian ALOX15 orthologs may also form dimers in aqueous solutions (76) the “Smith concept” might also be applicable for these enzymes (Fig. 4*B*), and the following hypothesis has been suggested: In the ALOX15 dimer, an allosteric regulator (activator or inhibitor) is bound in the substrate-binding pocket of the allosteric monomer (Fig. 5). This ligand binding induces conformational alterations in the structure of the allosteric monomer, which are further translated *via* intermonomer interactions to the catalytic monomer (Fig. 5). These structural changes may modify the substrate-binding properties of the catalytic monomer and thus may activate or inhibit fatty acid oxygenation. According to this concept, functional intermonomer communication (Fig. 4*B*) may be essential for the allosteric properties of mammalian ALOX15 orthologs.

To support this concept, the mode of action of substrate-specific ALOX15 inhibitors was recently explored. In wildtype human ALOX15, the intermonomer communication is functional, but in the Leu183Glu + Leu192Glu double mutant, it is defective (76). Thus, the Leu183Glu + Leu192Glu double mutant of human ALOX15 should have lost its allosteric properties. To test this hypothesis (89, 102), the IC_50_ values of substrate-specific ALOX15 inhibitors were determined. In these experiments, it was found that for wildtype human ALOX15, IC_50_ values for LA oxygenation were in the two-digit nanomolar range. (89, 102). When similar experiments were carried out with AA as substrate, the IC_50_ values were two orders of magnitude higher (89, 102). However, for the mutant enzyme, similar IC_50_ values (4.95 ± 0.38 μM for LA and 7.02 ± 0.91 μM for AA) were determined for the two substrates. These data suggested that the enzyme has lost its allosteric character when communication between the two monomers is disrupted. MD simulations of the enzyme–substrate–inhibitor complex of the ALOX15 dimer indicated that depending on the structure of the effector molecule, the substrate may adopt different conformations in the substrate-binding pocket of the catalytic monomer, which either improves or impairs the rate of initial hydrogen abstraction (89, 102).

Although the results of the substrate-specific inhibitor studies and the corresponding MD simulations suggest that ligand binding in the substrate-binding pocket of the allosteric monomer induces conformational changes in the catalytic monomer of the ALOX15 dimer, the detailed mechanisms and the kinetics of the ligand-induced intermonomer crosstalk remain elusive. The use of solution-based fluorine magnetic resonance spectroscopy, which was first applied for PTGS-isoenzymes, was an attempt to bridge the gap between static (situation in protein crystals) and dynamic (situation in aqueous solutions) information. Unfortunately, corresponding experiments have not been carried out for mammalian ALOX15 orthologs or for other ALOX-isoenzymes. Moreover, more detailed characterizations of the structural alterations involved in ligand-induced intermonomer communication require the application of additional solution-based technologies, such as FRET spectroscopy (103).

## Conflict of interest

The authors declare that they have no conflicts of interest with the contents of this article.
